# Coexistence of *Calliergonella cuspidata* and *Hamatocaulis vernicosus* Under Different Fen Topography Types and Microhabitat Conditions

**DOI:** 10.3390/plants15040651

**Published:** 2026-02-19

**Authors:** Monika Kalvaitienė, Ilona Jukonienė

**Affiliations:** State Scientific Research Institute Nature Research Centre, Akademijos St. 2, LT-08412 Vilnius, Lithuania; ilona.jukoniene@gamtc.lt

**Keywords:** Annex II of the Habitats Directive, fen management, hollows, hummocks, lawns, microtopography, moss transplantation, spring fens

## Abstract

*Hamatocaulis vernicosus* and *Calliergonella cuspidata* commonly co-occur in base-rich fens, reflecting overlapping ecological niches. While *C. cuspidata* is a widespread and ecologically plastic fen species often associated with eutrophicated wetlands, *H. vernicosus* is a habitat-specialist species of conservation concern. This study investigated the competitive interactions between these two moss species and the role of microhabitat conditions in their coexistence. A reciprocal transplant experiment was conducted in a natural, rich fen in southeastern Lithuania using replicated experimental plots across different microtopographic and hydrological conditions. Species cover and spread were monitored to assess competitive performance following transplantation. The results showed that under wet conditions, *H. vernicosus* was able to expand into surrounding areas and successfully compete with *C. cuspidata*. In contrast, *C. cuspidata* showed limited spread within *H. vernicosus* patches under wet conditions and was gradually displaced. An advantage of *C. cuspidata* was observed only in hummocky microtopographic settings. These findings indicate that stable hydrological conditions maintaining microhabitat heterogeneity promote the coexistence of both species. Alterations in the water regime may reduce the competitive ability and long-term persistence of *H. vernicosus*, highlighting the importance of hydrology-focused management for its conservation.

## 1. Introduction

Fens, which are characterised by high mineral concentrations arising from ground or surface water [1], are among the most species-rich and ecologically complex wetland habitats. Their topographic heterogeneity, expressed through microforms such as hummocks and hollows, creates spatial variation in hydrological, chemical, and microclimatic conditions [2], supporting a wide variety of bryophytes, including species of contrasting ecological requirements [3,4,5,6,7,8]. *Hamatocaulis vernicosus* (Mitt.) Hedenäs and *Calliergonella cuspidata* (Hedw.) Loeske are two moss species commonly found together in rich fens. However, they differ markedly in their ecological strategies, rarity, and sensitivity to environmental change.

*Hamatocaulis vernicosus*, a rare species of conservation priority, is protected under the Bern Convention [9] and listed in Annex II of the EU Habitats Directive [10], reflecting its vulnerability to environmental degradation, particularly eutrophication and hydrological fluctuations [11]. It typically occurs in mesotrophic and minerotrophic fens, which are characterised by stable hydrological regimes, high mineral availability, and minimal disturbance [11,12,13,14].

In contrast, *C. cuspidata* exhibits broad ecological tolerance and occurs across a wide range of habitats, including nutrient-rich or transitional fens [5,15]. Its ability to adapt morphologically in response to changes in light, moisture, and nutrient availability allows it to effectively colonise open or disturbed microhabitats [16]. The species is identified as a moss species that increases in abundance in fens undergoing early successional change triggered by nutrient enrichment [17,18,19,20]. Its expansion has been associated with the decline of calcicolous brown mosses, including *H. vernicosus*, particularly under elevated potassium or nitrogen levels [12,15,18,21].

The frequent occurrence of the competitive species *C. cuspidata* alongside the endangered European species *H. vernicosus* is believed to pose a significant threat to the latter [12,22]. Monitoring schemes were proposed that focus on interactions between *H. vernicosus* and *C. cuspidata*, as these may signal the initial phase of nutrient enrichment in seemingly intact fens within agricultural landscapes [15,22,23]. However, the experimental results show that increased N and P concentrations had a positive effect on the growth and biomass accumulation of the rare *H. vernicosus*, suggesting that nutrient availability alone does not fully explain competitive outcomes [24]. In this context, competitive outcomes between these species may also be shaped by fine-scale environmental heterogeneity, such as microtopography, which can modify local growing conditions independently of nutrient status. Despite its potential importance, the role of microrelief in mediating competition between *C. cuspidata* and *H. vernicosus* remains poorly understood. Field experiments with *H. vernicosus* demonstrated the species survival in the presence of *C. cuspidata,* depending on habitat conditions [25,26].

Since the habitats of *H. vernicosus* vary across Europe, it is essential to understand the role of competition in the local coexistence of these two species. Accurately interpreting monitoring signals and informing effective fen conservation and restoration strategies requires clarifying this relationship. Compared to many other regions of Europe, *H. vernicosus* habitats in Lithuania are diverse, occurring under a broader range of water chemistry conditions and with higher mineral content [14]. A key factor is the specific topography, which is characterised by shallow hollows and lawns fed by surface water that maintain a stable moisture regime. Lithuania stands out in Europe, having 37 Natura 2000 sites supporting *H. vernicosus* populations, as well as for the diversity of its habitats [27]. This highlights Lithuania’s significant responsibility for conserving this species on a European scale, and emphasises the importance of understanding the ecological requirements and potential threats facing *H. vernicosus* in this region.

Here we present the results of a transplant experiment conducted in the natural habitats of *H. vernicosus*, where the two species co-occur at equal abundance, forming distinct or mixed patches. During this study, we aimed: (1) to determine how species can survive when surrounded by patches of other species in different microhabitats; (2) to establish whether, once introduced, one species can successfully expand by replacing another species in its patches; and (3) to investigate the role of topographically driven microhabitat conditions in species competition.

This is important for predicting whether the growth of *C. cuspidata* alongside *H. vernicosus* could negatively impact *H. vernicosus*’s growth and survival across different fen topographies, and whether special management measures are always required to protect *H. vernicosus*.

## 2. Results

### 2.1. Changes in the Transplanted Subplots

#### 2.1.1. *Hamatocalis vernicosus*

**Figure 1:**
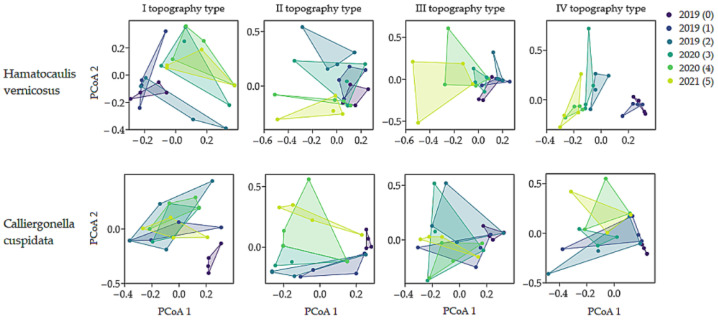
Temporal changes in transplanted *Hamatocaulis vernicosus* and *Calliergonella cuspidata* subplots across different topography types (I–IV) over the study period, visualised using PCoA ordination.

In the transplanted subplots of topography type I, the mean cover of *H. vernicosus* decreased from approximately 90% to 35.5% (Table 1). The PCoA ordination map reveals clearer separation of samples between 2019 and 2020–2021, indicating that population changes were more apparent over time rather than at early stages (Figure 1). The transplanted subplots of topography type II remained the most stable, with 41% of *H. vernicosus* coverage persisting. Although some fluctuations were observed in 2019, they did not persist in later years, reflecting a consistent, and relatively minor, level of change across the monitoring period (Figure 1). *Hamatocaulis vernicosus* in topography type III retained approximately 36% of mean cover, and subplots showed changes over the course of the study, with clearer differences becoming apparent at later stages. Topography type IV experienced the sharpest decline of *H. vernicosus* (Table 1). Changes in population structure are evident both in the early stages and in later years.

The changes in transplanted subplot structure varied greatly depending on the topography type, with the strongest structural divergence observed in types III and IV (Table 2, Figure 1). The cover of *Calliergonella cuspidata* increased in all transplanted *H. vernicosus* subplots (Table 1), although its contribution to structural changes—reflected in shifts in the proportional composition of subplot components such as *H. vernicosus* and herb covers —was most pronounced in topography type IV. In type I, it is likely that the dominance of *H. vernicosus* was mainly reduced by the amount of surface water. In type II, changes were minor, and *H. vernicosus* retained most of its coverage, showing structural stability. In type III, herb cover took over part of the former *H. vernicosus* location (Table 1).

#### 2.1.2. *Calliergonella cuspidata*

As in the case of *H. vernicosus* transplantation, the *C. cuspidata* transplanted subplot exhibited temporal changes (Figure 1). The most significant decline in *C. cuspidata* coverage was recorded in topography type I, where mean cover decreased to 17% (Table 3). There, changes were most apparent in the later stages of the study, with an increase in surface water area largely associated with the observed decline (Figure 1). In type II, coverage decreased from 97% to 36%, with significant differences observed between the early (2019) and later (2020–2021) periods, corresponding to increases in *H. vernicosus* and surface water (Figure 1, Table 3). In type III, coverage declined to 23%, reflecting strong sensitivity to the expansion of *H. vernicosus* and herbs. For topography type IV, coverage remained the highest, at about 50%, with the most noticeable changes occurring between early and later periods, mainly driven by an increase in herb cover. Overall, the dynamics of the transplanted *C. cuspidata* subplots were statistically significant, except for types III and IV, for which no consistent time effects were detected; however, multivariate dispersion analysis still revealed increased subplot heterogeneity after transplanting (Table 2).

### 2.2. Changes in the Surrounding Subplots after Transplantation

**Figure 2:**
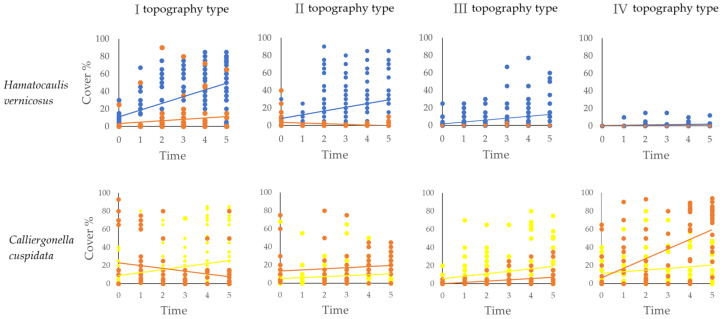
Temporal changes in transplanted species spread in surrounding subplots under experimental and control conditions (control shown in red) across different topography types (I–IV).

After *H. vernicosus* transplantation, a statistically significant positive trend was recorded in surrounding subplots of all topography types (Mann–Kendall test *p* ≤ 0.05) (Table 4). The most significant absolute increase (ΔP) was recorded in I (36.6%), and the smallest in IV (1.12%) topography type. Meanwhile, no significant changes were observed in the control subplots (*p* ≥ 0.5), and ΔP ranged from −5.66 to 4.18, showing only minor or negative changes (Figure 2).

*Calliergonella cuspidata* also showed a consistent, statistically significant positive trend in the surrounding fields after transplantation across all I–IV topography types (Table 4). The absolute change (ΔP) was highest in the surrounding subplots of IV (22.66%) and lowest in II (2.93%) topography type. A significant positive trend was also observed in the surrounding subplots for topography types II–IV, with ΔP ranging from 5.20 to 66.17% in type IV. Only in the I topography type control subplots change was negative (ΔP = −10.54), and the trend was statistically insignificant (*p* = 0.33). This shows that, except for topography type I, this species exhibited strong growth under both transplantation and control conditions, and this was especially evident in topography type IV, where the change was most significant.

## 3. Discussion

Our research focuses on the relationship between *C. cuspidata* and *H. vernicosus* within the context of related studies. *Calliergonella cuspidata* is often considered a competitively superior species when it co-occurs with *H. vernicosus*, which may contribute to the rarity and threatened status of the latter [22]. This interpretation is particularly applicable to eutrophicated habitats as *C. cuspidata* seemed to have an especially advantageous position as a nutrient-demanding species [12,15,18,21].

Our research differs from previous experimental studies primarily because it was conducted under natural field conditions in a relatively undisturbed rich fen. We focused on changes in local topography and microhabitat conditions, which ranged from flooded lawns and low hummocks to more elevated hummocky surfaces. The hollow-hummock gradient seemed to be important not only for the distribution of *H. vernicosus*, as shown in our previous study [14], but also for its competitive traits. Our recent results show that flooded lawns and low hummocks surrounded by spring flushes represent the most suitable microhabitats for *H. vernicosus*. These conditions not only support the persistence of the species but also facilitate its survival and dispersal once established within *C. cuspidata* patches. Although the abundance of *H. vernicosus* declined in transplanted subplots under highly flooded conditions, this reduction is more likely attributable to excessive hydrological stress than to competitive suppression. On the other hand, its expansion in the surrounding subplots was the highest among all topographical types. This interpretation is consistent with its known flood tolerance and previous observations of successful establishment and spread under inundated conditions [12,28]. In contrast, *C. cuspidata* showed limited performance under similar conditions, indicating lower tolerance to prolonged inundation. Our research further supports the conclusion of experimental laboratory studies that *H. vernicosus* is not a weaker competitor and may even outperform *C. cuspidata* in flooded conditions [29], suggesting that optimal water level, rather than interspecific competition, is the primary driver shaping *H. vernicosus* growth. Firstly, it keeps the vascular plant cover low [12], without reducing the solar radiation available to bryophytes. Secondly, spring waters create cool conditions, which also enhance the development of *H. vernicosus* as demonstrated in laboratory experiments [29]. The species also appears adapted to elevated concentrations of calcium (Ca^2+^), iron (Fe^2+^), and manganese (Mn^2+^), enabling it to form dense lawns just above the groundwater level rather than on higher hummocks [24,30]. *C. cuspidata*—like several morphologically and phylogenetically distinct species, such as *Aulacomnium palustre*, *Helodium blandowii* and *Marchantia polymorpha*—shares intermediate hummock-forming frequency and desiccation avoidance. The importance of suitable fen conditions. These ecological requirements are consistent with observations from *Hamatocaulis vernicosus* reintroductions, where successful establishment occurred only under suitable fen conditions [25,26].

It is important to note that, despite being in control plots without transplantation, expansion of both *H. vernicosus* and *C. cuspidata* was observed, and the absolute numbers, with the exception of those for *C. cuspidata* expansion in hummocky areas, were not significant. Apparently, over a more extended period as they grow together, these species reach equilibrium, and changes in their abundance are not significant; however, in the case of disturbances, *H. vernicosus* has the competitive advantage.

It is important to note that, although expansion of both *H. vernicosus* and *C. cuspidata* was observed during the study period, their responses differed substantially between microhabitats. The expansion of *H. vernicosus* was most pronounced in the lawn and other non-hummock areas associated with higher groundwater levels and was largely restricted to transplanted subplots, with minimal change in hummocky conditions. This indicates that the observed increase in *H. vernicosus* was driven by transplantation and constrained by drier, elevated microhabitats. In contrast, *C. cuspidata* showed robust expansion in hummocky areas, including substantial increases in control plots. Overall, these results demonstrate species-specific and topography-dependent expansion patterns, with *H. vernicosus* responding primarily to transplantation and *C. cuspidata* exhibiting high ecological plasticity.

Our experiments show that *C. cuspidata* has a competitive advantage over *H. vernicosus* only in hummocky areas. Hummock microtopography establishes specific microclimatic conditions, with small-scale variations in soil thermal properties and water regimes [31]. Due to hummocks, bryophytes are distant or isolated from mineral fen water [32,33]. In the absence of cold spring water, bryophytes experience warmer microclimatic conditions. So, our results are in accordance with laboratory experiments showing that warmer environments are favourable for the growth of *C. cuspidata* [29]. Although *H. vernicosus* decreased significantly in drier conditions in recent years of the experiment compared to the first years, this decrease was followed not only by an increase in *C. cuspidata*, but also by the growth of herbs. It is unlikely that *H. vernicosus* and *C. cuspidata* can also benefit from the increased shading that results from the enhanced growth of vascular plants [34]. We did not analyse habitat eutrophication. Based on research in hummocky areas, we cannot confirm or deny whether *C. cuspidata* is competitively stronger than fen moss specialists when nutrient supply is increased [18] because nutrient cycling in hollow-hummock microtopography is different. Some studies show that drier environments and hummocks in fens typically have faster nutrient cycling [35] and nitrogen release [36]. In fen systems, hydrological gradients associated with microtopography strongly influence nutrient availability and plant performance, often overriding nutrient enrichment effects observed at the habitat scale [15,28].

Although our studies covered natural fen with changing topographic types, it is important to note that the expansion of drier areas can be accelerated by human activity [1]. *H. vernicosus* displays fragmented and human-sensitive populations throughout Europe, with its local distribution being strongly influenced by wetland hydrology [37]. Drainage of wetlands enhances the processes when, similar to the formation of hummocks, the top peat layer loses contact with mineral-rich groundwater. Drained fens develop more homogenised areas dominated by dry-adapted mosses, instead of heterogenic fen areas with changing hummock-hollow microtopography [30]. In such conditions, *C. cuspidata* will have an advantage over *H. vernicosus*, as stated in hummocky areas by us.

## 4. Materials and Methods

### 4.1. Study Site and Experimental Design

**Figure 3:**
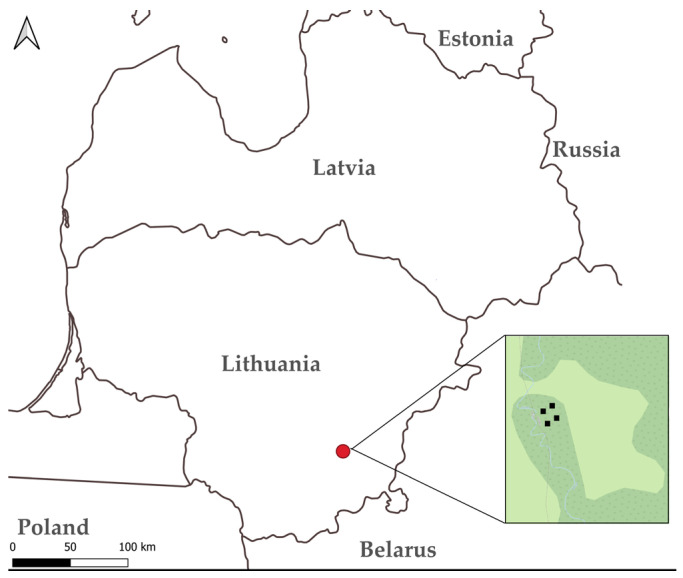
Map of study site (red dot) and four study areas (black squares).

The experimental study was conducted at the Bražuolė fen, in the Trakai district, Lithuania (Figure 3). This site is situated along the right bank of the Bražuolė River and represents a sparsely populated area characterised by low-intensity land use. The fen supports naturally occurring populations of *H. vernicosus*, making it a suitable location for transplantation and monitoring studies. The habitat is predominantly wet, with high groundwater levels and a mosaic of hummock, hollow and lawn microtopographies, providing a range of microhabitats relevant for assessing species-specific expansion patterns. Its relatively undisturbed condition allows for evaluation of natural responses to experimental interventions without substantial confounding anthropogenic impacts.

**Figure 4:**
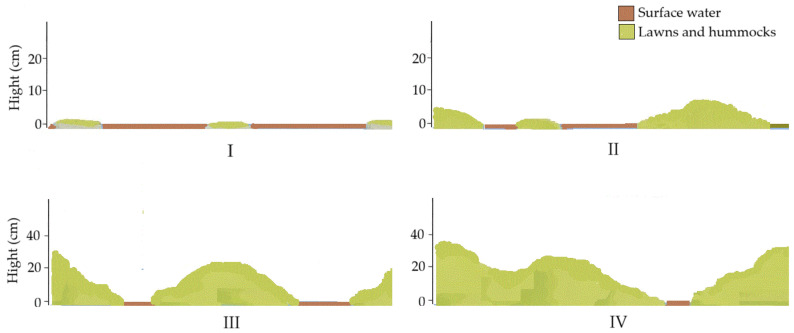
Schematic representation of topography types I–IV.

The research was carried out from 2019 to 2021. Four study areas were selected within the fen to represent different topographical and hydrological conditions across the site (Figure 4). Two study areas were dominated by bryophyte lawns associated with a consistently high groundwater table; one study area exhibited flat, continuous bryophyte lawns up to two-thirds submerged in water (topography type I), whereas the other—displayed abundant surface, interspersed with bryophyte lawns just above the surface water or low hummocks (5–10 cm high) (topography type II). The remaining two sites were characterised by hummock–hollow microtopography, with more elevated, densely vegetated hummocks. One of these hummocky sites exhibited water-filled interhummock hollows (topography type III), whereas the other displayed comparatively drier interhummock areas with limited surface water presence (topography type IV). The selected study areas reflected a range of typical fen microhabitats, from wetter, more open lawns to drier, more hummock-dominated zones, capturing the natural heterogeneity of fen ecosystems. The characteristics of each study area are summarised in Appendix A. In each study area, 12 long-term study plots were established, with stakes placed at the corners to mark plot boundaries for future identification. The plots were categorised as follows: (a) four plots dominated by *H. vernicosus*, (b) four plots dominated by *C. cuspidata*, (c) four control plots: two of which were dominated by *H. vernicosus*, and two by *C. cuspidata*. The study plots covered different microtopography types, where both species were present: submerged lawns (topography type I), lawns or low hummocks above water level (topography type II), hummocks surrounded by spring waters (topography type II) and comparatively dry interhummock areas (topography type IV). In total, 48 study plots were created across all areas. Each study plot measured 30 × 30 cm and was divided into nine equal subplots of 10 × 10 cm. Within each subplot, the following parameters were recorded:

(a) Total herb cover (%);
(b) Surface water cover (%);
(c) Total bryophyte cover (%);
(d) Species composition of all bryophytes and their individual cover (%).

**Figure 5:**
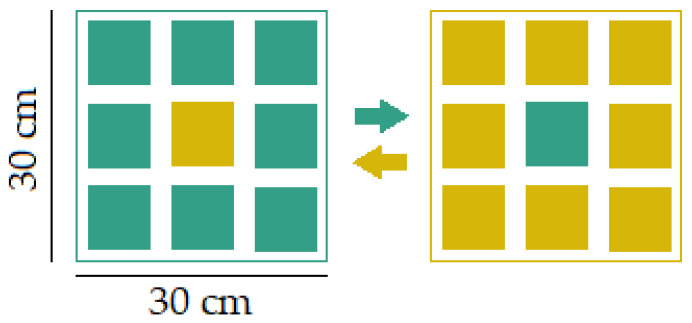
Reciprocal transplant design. The central 10 × 10 cm^2^ subplot was transplanted between plots dominated by species *Hamatocaulis vernicosus* (green squares) and species *Calliergonella cuspidata* (yellow squares). Arrows indicate reciprocal exchange of the central subplot.

A total of 432 subplots were assessed across all study plots. The experimental design involved transplanting vegetation from the central subplot of each study plot to the central subplot of a plot dominated by the opposite species, creating a reciprocal transplant between *H. vernicosus* and *C. cuspidata* (Figure 5). Specifically, a central subplot from a study plot dominated by *H. vernicosus* was moved to a study plot dominated by *C. cuspidata*, and vice versa. The study plots were monitored from 2019 to 2021. The experimental plots were initially planted in April, with subsequent observations and recordings carried out in June and September of the same year. In 2020, additional monitoring took place during spring and the second half of summer, and in spring 2021. In order to compare temporal changes in the abundance of *C. cuspidata* and *H. vernicosus* under natural conditions, transplantation was not carried out in the control plots.

### 4.2. Data Analysis

Changes were assessed in transplanted subplots and in surrounding subplots, and these were compared with subplots from control study plots. Separate analyses were conducted to evaluate changes within the central transplanted subplots. Additional analyses assessed whether the introduced species altered adjacent plots relative to the controls.

The PERMDISP test based on Bray–Curtis dissimilarities was used to assess whether the dispersion of transplanted *H. vernicosus* and *C. cuspidata* subplots across different topographic types was homogeneous. Distances to group centroids were calculated for each topography type, and significance was tested using 999 permutations. To evaluate differences in subplot composition (covering all components of the transplanted subplot) in the topographic types studied, a one-way PERMANOVA analysis was performed. The analyses were performed using the Bray–Curtis dissimilarity measure. A SIMPER (Similarity Percentage) analysis was performed to determine which components most influenced the changes in the transplanted subplot during the study period. Community compositional changes were quantified using the Bray–Curtis dissimilarity index based on percentage cover data of herbs, surface water, and individual moss species. Only those components whose cumulative contribution (cumulative %) accounted for up to 80% of the total dissimilarity were used for the analysis.

In addition, to illustrate differences in community structure, principal coordinate analysis (PCoA) plots were generated to depict the distribution of subplots across different types of topography during the study period.

The Mann–Kendall test was applied to test for temporal trends in the abundance of the transplanted species increased in the surrounding subplots following transplantation to the central subplot. To quantify the magnitude of change in *H. vernicosus* and *C. cuspidata*, absolute cover change was calculated as Δcover (ΔP) = final cover − initial cover, and the same procedure was applied to control subplots for comparison. A regression curve was used to visually display temporal changes in species cover in both the surrounding and control subplots, enabling direct comparison of trends between the experimental and control conditions.

All statistical analyses and graphical visualisations were conducted using PAST statistical software, version 5.3 [38], and additional figures were created in Microsoft Excel. Any *p*-values less than 0.05 were regarded as statistically significant.

Taxonomic nomenclature followed the checklist of bryophytes of Europe [39] and the World Checklist of Vascular Plants [40].

## 5. Conclusions

Our studies, conducted in topographically and micro-relief-wise different areas of the same fen that are not affected by external nutrient enrichment from the surrounding area, demonstrate the significant role of environmental condition alterations within fens in preserving and maintaining *H. vernicosus* populations. If the water regime maintains heterogeneity in fen microhabitats, *C. cuspidata* and *H. vernicosus* can coexist successfully without additional management to increase the abundance of the latter. This is especially true in flooded areas dominated by lawns or low hummocks compared to hummocky areas. However, the abundance and competitive characteristics of *H. vernicosus* may decline with changes in the water regime. Implementing supplementary management measures is solely necessary in fens where drier, hummocky areas are naturally or human-impact-dominated.

## Figures and Tables

**Table 1:** Components contributing most to cover change in *Hamatocaulis vernicosus* transplanted subplots in topography types I–IV * (see Materials and Methods section, as well as Appendix A, for more detailed information). Only components whose cumulative contribution accounted for up to 80% of the total Bray–Curtis dissimilarity are included.

Topography Type	Cover	Cumulative %	Initial Mean Cover %	Final Mean Cover %
I	*H. vernicosus*	33.45	90	35.5
	Surface water	56.49	2.5	25
	*C. cuspidata*	76.11	4	30
II	*H. vernicosus*	42.95	88.8	40.8
	*C. cuspidata*	61.27	1.75	22
	Herb	76.81	15	32
III	Herb	32.41	5	50
	*H. vernicosus*	61.76	74.8	36.3
	*C. cuspidata*	78.51	15.3	28
	Surface water	89.33	0	15
IV	*H. vernicosus*	40.15	88.3	4.25
	*C. cuspidata*	70.87	1	66
	Herb	89.54	4.75	44.5

* Topography types: I—wet bryophyte lawns (surface water up to 70%), II—low hummocks (surface water up to 50%), III—hummocks with surface water up to 30%, IV—hummocky area (surface water negligible).

**Table 2:** Summary of Multivariate dispersion and one-way PERMANOVA test in transplanted *Hamatocaulis vernicosus* subplots in different topography types.

Test	Topography Type	*Hamatocaulis vernicosus*			*Calliergonella cuspidata*		
		F	R^2^	*p*	F	R^2^	*p*
Multivariate dispersion	I	3.53	-	0.02	1.75	-	0.13
	II	2.44	-	0.06	3.21	-	0.03
	III	17.45	-	0.00	3.74	-	0.02
	IV	5.57	-	0.00	4.67	-	0.00
PERMANOVA	I	2.56	0.42	0.02	2.9	0.45	0.00
	II	1.73	0.33	0.07	3.02	0.46	0.00
	III	3.23	0.47	0.00	1.88	0.34	0.06
	IV	3.23	0.47	0.00	1.79	0.35	0.10

**Table 3:** Components contributing most to cover change in *Calliergonella cuspidata* transplanted subplots in topography types I–IV (see Materials and Methods section, as well as Appendix A, for more detailed information). Only components whose cumulative contribution accounted for up to 80% of the total Bray–Curtis dissimilarity are included.

Topography Type	Cover	Cumulative %	Initial Mean Cover %	Final Mean Cover %
I	*C. cuspidata*	48.63	92	17.5
	Surface water	77.69	0	47
	Herb	89.44	11.8	30
II	*C. cuspidata*	43.28	97.3	36
	*H. vernicosus*	63.87	2	31
	Surface water	83.37	0	27.5
III	*C. cuspidata*	41.32	92.3	23.8
	*H. vernicosus*	61.6	2.5	35
	Herb	80.42	7.5	38.8
IV	*C. cuspidata*	44.22	99.5	49.5
	Herb	76.03	2	41.3
	Surface water	85.87	0	11.3

**Table 4:** Mann–Kendall test results and absolute change (ΔP) in the spread of introduced species into neighbouring plots across control and introduction treatments.

Introduced Species	Subplot Type	Statistical Parameters	Topography Type			
			I	II	III	IV
*Hamatocaulis vernicosus*	Treatment	S	369	1995	1952	620
		Z	2.13	2.78	2.36	2.83
		*p*	0.03	0.00	0.01	0.00
		ΔP	36.6	21.47	6.89	1.12
	Control	S	260	−291	16	0
		Z	0.97	−1.10	0.16	0
		*p*	0.32	0.27	0.87	1
		ΔP	4.18	−5.66	0	0
*Calliergonella cuspidata*	Treatment	S	1924	2706	3551	1700
		Z	2.32	3.32	4.85	2.96
		*p*	0.01	0.00	0.00	0.00
		ΔP	10.96	2.93	15.31	22.66
	Control	S	−286	829	991	1438
		Z	−0.96	3.05	3.04	4.22
		*p*	0.33	0.00	0.00	0.00
		ΔP	−10.54	5.20	6.88	66.17

## Data Availability

The data presented in this study are available upon request from the corresponding author. The data are not publicly available due to privacy concerns.

## Appendix A. Characteristics of the Study Areas

**Study Area**	**1**	**2**	**3**	**4**
Open water (%)	70	50	30	5
Herb cover (%)	25	30	70	80
Hummock cover (%)	30	50	60	70
Bryophyte cover (%)	30	50	80	90
**Herbs**				
*Carex rostrata*	3	2	3	3
*Cardamine palustris*	1	1	1	1
*Rumex acetosa*	1	1	1	2
*Eriophorum angustifolium*		1	2	2
*Festuca rubra*	1	1	1	
*Silene flos-cuculi*		+	2	2
*Calla palustris*		+	1	
*Galium uliginosum*		1		1
*Carex diandra*			1	
*Menyanthes palustris*			3	
*Caltha palustris*		1		
*Equisetum fluviatile*				1
*Lemna minor*		1		
*Typha latifolia*			1	
**Bryophytes**				
*Calliergonella cuspidata*	2	2	3	3
*Hamatocaulis vernicosus*	2	3	2	2
*Ptychostomum pseudotriquetrum*	1	1	1	2
*Aulacomnium palustre*		1	1	2
*Plagiomnium ellipticum*		1	2	2
*Tomentypnum nitens*		1	1	2
*Brachythecium mildeanum*			2	2
*Marchantia polymorpha*	1	1		
*Calliergon giganteum*	1			
*Helodium blandowii*			2	
**Water physical and chemical parameters:**				
pH	6.68	6.8	6.92	7.2
Conductivity (µS/cm)	370	332	313	327
Ca^2+^ (mg/L)	73.7	71.7	66.1	64.5
Fe^3+^ (mg/L)	4.86	8.11	7.26	3.93
K^+^ (mg/L)	4.49	3.33	1.31	0.80
Mg^2+^ (mg/L)	23.4	28.5	24.6	19.7
NH_4_^+^ (mg/L)	0.06	0.15	0.16	0.02
NO_3_^−^ (mg/L)	0.04	0.06	0.01	0.1
PO_4_^3−^ (mg/L)	0.03	0.13	0.05	0.15
